# United States College of Veterinary Surgeons

**Published:** 1899-05

**Authors:** 


### UNITED STATES COLLEGE OF VETERINARY SURGEONS.

The commencement exercises of the college were held in the lecture hall of the college on Thursday, April 13th. Dr. C. Barnwell Robinson opened the exercises with an address, after which he conferred the degrees of doctor of veterinary science upon the following graduates: E. P. Flower, New Orleans, La.; J. G. Ferneyhough, Blacksburg, Va.; C. E. Uber, Glencarlyn, Va. The degree of fellowship was conferred upon Professors H. D. Hanson, D.V.S., of New York City, and Otto G. Noack, veterinarian, of Reading, Pa.

The prizes were then awarded as follows: Mr. E. Pegram Flower, of New Orleans, La., the Hinkley prize for general proficiency and the Gheen prize for practice of medicine and surgery; Mr. Frank B. Berger, of Baltimore, Md., the gold medal presented by Dr. William E. Yetton for proficiency in dentistry; Mr. J. G. Ferneyhough, the second prize for general proficiency, and Mr. Frank F.

Feagons, of West Virginia, the Kaufmann prize, a library of books, for the best junior examination.

The trustees were represented by Prof. George A. Prevost, who in a few remarks explained the close relation which the veterinary profession held to society, not only in reference to treatment of animals when diseased or injured, but as to public health in meat-inspection and milk-inspection, and said the army scandal could have been avoided if the veterinarians had been called into service at the outbreak of the late war with Spain. He was followed by Mr. Flower, the valedictorian of the class of 1899, who delivered an address which was received with great applause. This was responded to by Prof. Robert S. Lamb, M.D., representing the Faculty. He spoke of the relation existing between students and professors, and admonished the former to always remember their alma mater. This address was followed by that of Prof. W. A. Hedrick, Ph.D., who presented the humorous side of the many problems confronting the students in the career presented by veterinary science.

The Fellows of the college were represented by Dr. Otto G. Noack, who drew the attention of the graduates to the fact that the "golden rule" should always be observed by them; that diagnosis, prognosis, and treatment should be governed by the marked conditions present. He also admonished the graduates to endeavor to advance the profession by word and deed whenever the opportunity presented itself.

The class of 1898 was represented by Dr. George W. Bready, of Maryland, who described the trials and tribulations of the young practitioner.

The exercises were closed by Dr. Robinson, who thanked the friends of the college for their many valuable donations during the past year, and spoke of the growth and prosperity of the institution.

